# A Case of Colon Cancer-Induced Hemophagocytic Lymphohistiocytosis with Lymphangitic Carcinomatosis

**DOI:** 10.70352/scrj.cr.26-0163

**Published:** 2026-06-09

**Authors:** Ayaka Tachikawa, Kazushige Kawai, Akira Dejima, Akira Sakamoto, Sakiko Nakamori, Hiroki Kato, Misato Takao, Daisuke Nakano

**Affiliations:** Department of Colorectal Surgery, Tokyo Metropolitan Cancer and Infectious Diseases Center, Komagome Hospital, Tokyo, Japan

**Keywords:** colorectal cancer, hemophagocytic lymphohistiocytosis

## Abstract

**INTRODUCTION:**

Hemophagocytic lymphohistiocytosis (HLH) is a syndrome involving extreme hyperinflammation driven by a cytokine storm, which leads to the pathological autophagocytosis of blood cells. Although secondary HLH is frequently associated with underlying malignancies, the majority of which are hematological, cases triggered by colorectal cancer are quite rare. We report herein a case of HLH induced by ascending colon cancer.

**CASE PRESENTATION:**

An 83-year-old female patient presented to her primary care physician with a 1-month history of anorexia, persistent fever (>38°C), and cognitive decline. Colonoscopy revealed a tumor at the hepatic flexure of the ascending colon, which was identified as a well-differentiated adenocarcinoma on the basis of a biopsy specimen analysis. Ascending colon cancer (cT3N2bM0, cStage IIIc) was finally diagnosed. As the fever was presumed to be a paraneoplastic manifestation, the patient underwent robotic-assisted right hemicolectomy with lymphadenectomy. Recurrences of the fever, persistent anorexia, and generalized edema were observed postoperatively. On POD 25, peripheral blood smears revealed macrophage-mediated hemophagocytosis, confirming the diagnosis of HLH based on the HLH-2004 diagnostic criteria. Despite an escalation in corticosteroid therapy, the patient remained refractory to treatment and died on POD 46.

**CONCLUSIONS:**

Clinicians should include cancer-induced HLH in the differential diagnosis of fever and bicytopenia in the presence of an advanced solid tumor.

## Abbreviations

DIC
: disseminated intravascular coagulation
HLH
: hemophagocytic lymphohistiocytosis
ICI
: immune checkpoint inhibitor
M-HLH
: malignancy-associated HLH
NK
: natural killer
OR
: odds ratio
sIL-2R
: soluble interleukin-2 receptor

## INTRODUCTION

HLH is a syndrome involving extreme hyperinflammation driven by a cytokine storm, which leads to the pathological autophagocytosis of blood cells. The condition is categorized into familial and secondary forms; the former occurs in more than 60% of patients by the age of 3 years,^1)^ whereas the latter typically appears around the age of 50 years.^2)^ No sex difference has been observed. Secondary HLH is frequently associated with an underlying infection, malignancy, or autoimmune disease.^3,4)^ M-HLH has an exceptionally poor prognosis: the median survival time is approximately 2 months, and the 1-year survival rate is lower than 20%. Therefore, early detection and prompt treatment are crucial for improving outcomes.^3,5)^ While hematological malignancies account for the majority of M-HLH cases, solid tumors represent less than 3% of cases.^3,4)^ Cases induced by colorectal cancer are especially rare. We report herein a case of HLH that was diagnosed following the surgical resection of an ascending colon cancer.

## CASE PRESENTATION

**Fig. 1:**
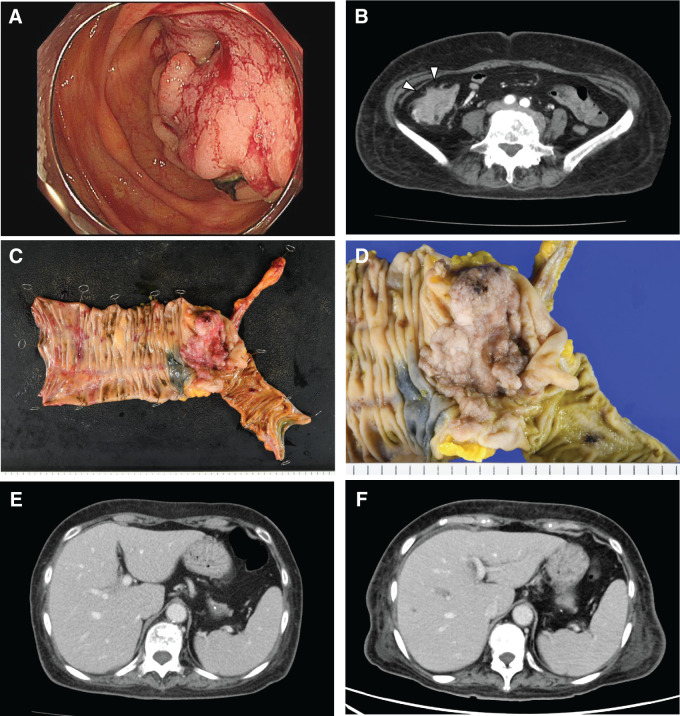
Imaging studies and resected specimen. (**A**) Lower gastrointestinal endoscopy findings. A tumor measuring 60 mm was observed in the ascending colon. (**B**) Contrast-enhanced CT findings. A tumor with enhancement measuring 60 mm was observed in the ascending colon (white arrowheads) with multiple lymphadenopathies, but there was no clear evidence of a distant metastasis. (**C**, **D**) A surgical specimen from the ascending colon revealed a tumor measuring 60 × 40 mm. (**E**, **F**) A retrospective review of the preoperative CT findings revealed that the longitudinal diameter of the spleen was 104 mm (**E**), which increased to 111 mm on POD 8 (**F**), suggesting mild splenomegaly.

An 83-year-old female patient presented to her primary care physician with a 1-month history of anorexia, persistent fever (>38°C), and cognitive decline. A thorough evaluation revealed ascending colon cancer, and she was referred to our center for surgery. Colonoscopy showed a tumor at the hepatic flexure of the ascending colon, and an analysis of a biopsy specimen confirmed the presence of well-differentiated adenocarcinoma (**Fig. 1A**). Contrast-enhanced CT demonstrated a thickened colonic wall at the same site, increased peritumoral fat density, and multiple lymphadenopathies (**Fig. 1B**). No distant metastases were identified, and no definitive infectious focus explaining the fever was found. Based on these findings, ascending colon cancer (cT3N2bM0, cStage IIIc) was diagnosed. Preoperative laboratory tests revealed bicytopenia (hemoglobin, 7.4 g/dL; platelets 9.2 × 10^4^/μL), ferritin 529.4 μg/L, sIL-2R 4996 U/mL, triglycerides 152 mg/dL, and fibrinogen 544 mg/dL. The fever was presumed to be a paraneoplastic manifestation; thus, the patient underwent robot-assisted right hemicolectomy with lymphadenectomy. The operation lasted 3 h 14 min, and the total blood loss was 10 mL. The resected specimen revealed a 60-mm tumor in the ascending colon (**Fig. 1C** and **1D**). Postoperatively, fever recurred, and the patient had persistent anorexia and generalized edema. On POD 8, CT revealed bilateral pulmonary infiltrates and cardiomegaly, based on which bilateral pneumonia and acute heart failure were diagnosed. Antibiotic therapy and diuretic administration were initiated. While peripheral blood smears revealed atypical lymphocytes, cerebrospinal fluid analysis conducted to assess for lymphoma yielded unremarkable findings. Dexamethasone therapy was begun on POD 14 for diagnostic and therapeutic purposes, but the clinical response was poor. On POD 25, peripheral blood smears showed macrophage-mediated hemophagocytosis. In addition to persistent fever, laboratory tests revealed bicytopenia (hemoglobin, 8.7 g/dL; platelets, 4.7 × 10^4^/μL) and markedly elevated ferritin (2538.7 μg/L) and sIL-2R (9270 U/mL).

**Fig. 2:**
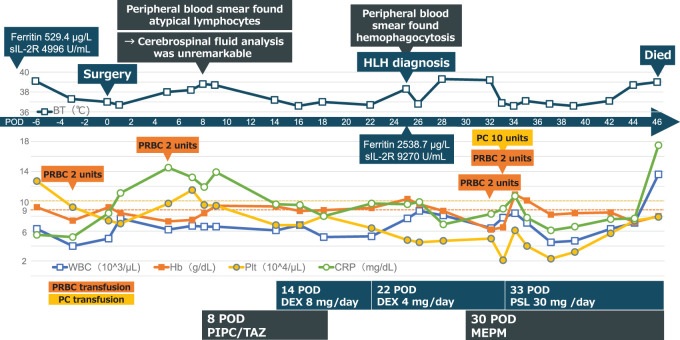
Postoperative clinical course. On POD 8, a peripheral blood smear found atypical lymphocytes, while a cerebrospinal fluid analysis was unremarkable. However, on POD 25, a peripheral blood smear demonstrated hemophagocytosis. Based on the presence of fever, splenomegaly, cytopenia affecting 2 lineages, elevated ferritin, and increased sIL-2R level, HLH was finally diagnosed. The diagnostic criteria for cytopenia in HLH include hemoglobin <9 g/dL and platelets <100 × 10^9^/L. BT, body temperature; CRP, C-reactive protein; DEX, Dexamethasone; Hb, hemoglobin; HLH, hemophagocytic lymphohistiocytosis; MEPM, meropenem; PC, concentrated platelets (10 unit = 400 mL); PIPC, piperacillin; Plt, platelet; PRBC, packed red blood cells (2 unit = 240 mL); sIL-2R, soluble interleukin-2 receptor; TAZ, tazobactam; WBC, white blood cell

A retrospective review of the preoperative CT findings found the spleen to have a longitudinal diameter of 104 mm, which increased to 111 mm on POD 8, suggesting mild splenomegaly (**Fig. 1E** and **1F**).^6)^ HLH was ultimately diagnosed on the basis of the HLH-2004 diagnostic criteria^7)^ (**Fig. 2**). Despite an escalation in the corticosteroid dosage, the patient remained refractory to treatment and died on POD 46.

**Fig. 3:**
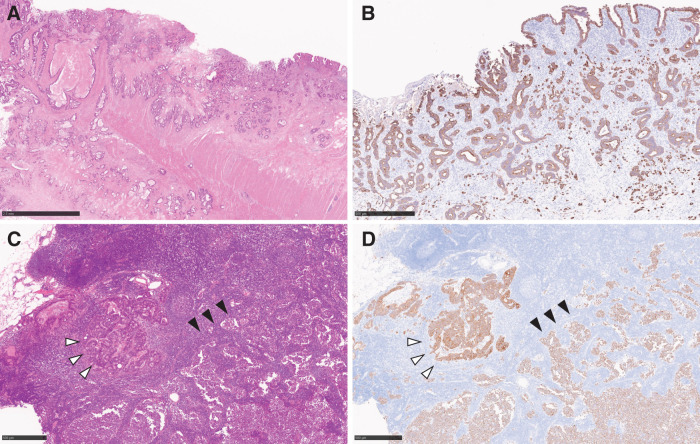
Histopathological findings. (**A**, **C**) HE staining. (**B**, **D**) Immunohistochemically, the tumor cells were diffusely positive for CAM5.2 (brown). (**A**, **B**) The resected colorectal tumor contained components consisting predominantly of moderately differentiated tubular adenocarcinoma 2 (tub2) with focal tub1 and poorly differentiated adenocarcinoma 1 (por1). (**C**, **D**) Dissection of the 42 lymph nodes assessed found a metastasis consistent with moderately to well-differentiated adenocarcinoma in 6 (white arrowheads). Histological analysis of the remaining 27 nodes demonstrated a distinct pattern of poorly differentiated carcinoma with low cell adhesion occupying the lymphatic sinuses, unlike the differentiated adenocarcinoma (black arrowheads). HE, hematoxylin–eosin

Histopathological analysis of the resected colon cancer specimen determined that the final stage was pT4aN2b, pStage IIIc (**Fig. 3A** and **3B**). A dissection of 27 lymph nodes found poorly differentiated carcinoma with diminished cell adhesion filling the lymphatic sinuses and having a histological appearance distinct from that of the primary lesion, indicating lymphangitic carcinomatosis (**Fig. 3C** and **3D**). Hemophagocytic findings of the lymph nodes were unremarkable.

## DISCUSSION

HLH is a syndrome involving extreme hyperinflammation driven by a cytokine storm, which leads to the pathological autophagocytosis of blood cells. It is diagnosed using the HLH-2004 criteria. Cases stemming from genetic mutations are classified as familial HLH; secondary HLH is diagnosed when a patient satisfies at least 5 of the following 8 clinical parameters: 1. fever; 2. splenomegaly; 3. cytopenia affecting ≥2 peripheral blood lineages (hemoglobin, <9.0 g/dL; platelets, <10 × 10^4^/μL; neutrophils, <1000/μL); 4. hypertriglyceridemia (>265 mg/dL) and/or hypofibrinogenemia (<150 mg/dL); 5. hemophagocytosis in bone marrow, the spleen, or lymph nodes; 6. low or absent NK cell activity; 7. hyperferritinemia (>500 ng/mL); and 8. elevated sIL-2R (>2400 U/mL).^7)^

The etiology of HLH frequently involves an underlying infection, autoimmune disease, or malignancy, particularly hematological malignancies.^3,4)^ The condition manifests as a cytokine storm driven by excessive immune activation. A persistent presentation of antigens and sustained immunological stimulation can induce aberrant activation of antigen-presenting cells, thereby amplifying the release of inflammatory cytokines. This cascade activates cytotoxic T lymphocytes and NK cells, with cytokines such as interferon-γ further potentiating macrophage activation. This, in turn, creates a positive feedback loop of immune activation, often mediated by the JAK-STAT signaling pathway, which ultimately results in organ injury. For these reasons, administering an appropriate treatment promptly before progression to multiorgan failure is critical for improving outcomes.^8,9)^ Indeed, a retrospective cohort study by Abdelhay et al. involving adult HLH patients demonstrated that delays in treatment initiation were associated with higher in-hospital mortality (OR: 2.0).^10)^ Circulatory shock (OR: 1.33) and mechanical ventilation (OR: 1.41) were also more prevalent in the delayed-treatment cohort.

M-HLH induced by a solid tumor is uncommon.^3,4)^ HLH associated with colorectal cancer is especially rare, with a review of the relevant literature to date identifying only 8 such cases (**Table 1**), many of which were triggered by a concurrent autoimmune disease,^11)^ anticancer chemotherapy,^12,13)^ ICI therapy,^11,14)^ or infection.^15,16)^ The present case is apparently only the third of its kind after those reported by Ali and Debbarma, and by Jiménez Rosales et al., where the colorectal malignancy itself served as the primary driver of the syndrome.^17,18)^ A distinctive feature of our case was the marked lymphatic invasion by highly aggressive tumor cells, as confirmed by postoperative pathological analysis. It is highly probable that the advanced malignancy amplified immune activation and contributed to HLH development via a cytokine storm. However, future studies might elucidate the mechanism of HLH development in highly aggressive malignancies, such as the present one.

**Table 1:** HLH associated with colorectal cancer in PubMed

No.	Author	Year	Age	Sex	TNM	Stage	Surgery	Probable trigger	Autoimmune disease	Treatment	Outcome
1	Yamada et al.^15)^	2002	62	M	T4a N0 M0	IIB	+	Colon cancer/drug/intra-abdominal abscess	—	Systemic corticosteroid	HLH resolved
2	Oliveira et al.^16)^	2014	75	M	TND N0 M0	I	+	Infectious peritonitis	ND	Systemic corticosteroid	Death
3	Ali and Debbarma^17)^	2017	42	M	T3 N2b M0	IIIc	—	Colon cancer	—	Systemic corticosteroid + FOLFOX	HLH resolved
4	Jiménez et al.^18)^	2019	57	M	TND NND M1a	IV	+	Rectal cancer	—	Systemic corticosteroid	Death
5	Jaffrelot et al.^12)^	2021	65	F	TND NND M1a	IV	—	Regorafenib	ND	Discontinuation of regorafenib + systemic corticosteroids	HLH resolved
6	Blazevic et al.^13)^	2024	64	F	TND N+ M1c2	IV	+	Regorafenib	—	Discontinuation of regorafenib + systemic corticosteroids	HLH resolved
7	Jiao et al.^11)^	2025	68	F	TND N+ M+	IV	—	Tislelizumab	SLE	Discontinuation of ICI + systemic corticosteroids	Death
8	Lentz et al.^14)^	2025	ND	ND	TND NND M+	IV	ND	Evorpacept/cetuximab/pembrolizumab	ND	High-dose steroid + tocilizumab + mycophenolate mofetil	Death
9	Our case	2026	83	F	T4a N2b M0	IIIc	+	Colon cancer	—	Systemic corticosteroid	Death

HLH, hemophagocytic lymphohistiocytosis; ICI, immune checkpoint inhibitor; ND, no data; SLE, systemic lupus erythematosus

Diagnosing M-HLH remains a significant clinical challenge due to the substantial overlap in clinical manifestations and laboratory abnormalities of HLH and the malignancy, which often leads to diagnostic delays.^19)^ According to a recently published review, ICI-associated HLH is a rare but life-threatening syndrome stemming from immune activation induced by ICI therapy or drug hypersensitivity. As the use of ICIs for the treatment of solid tumors, including colorectal cancer, increases, the incidence of ICI-associated HLH is also likely to rise.^20)^ In the perioperative setting, fever is more commonly attributed to a surgical site infection, sepsis, DIC, or related complications; thus, establishing a diagnosis of HLH before hemophagocytosis becomes apparent on a peripheral blood smear is challenging. In this case, despite the preoperative presence of fever, secondary HLH was not definitively diagnosed until POD 25. Notably, a retrospective review of the preoperative CT findings revealed mild splenomegaly that had not been noted on the initial reading. If HLH had been considered in the differential diagnosis earlier, it might have been diagnosed sooner. Thus, in the presence of a fever and cytopenia affecting 2 or more lineages, clinicians should consider HLH early in the differential diagnosis and re-evaluate the laboratory parameters using the HLH-2004 diagnostic criteria, while reassessing peripheral blood and bone marrow specimens for hemophagocytic findings if clinically warranted.

Several promising therapeutic agents for HLH, including etoposide, intravenous immunoglobulin, and other immunosuppressive agents, such as emapalumab, have been reported. These agents might have been effective in this case,^21)^ but because postoperative HLH is difficult to distinguish from a fever or an infectious complication, the definitive diagnosis was delayed. As a result, by the time the disease was diagnosed, the patient’s systemic condition had already deteriorated to the point at which his consciousness status had become impaired and hepatic failure had developed, thereby precluding the administration of any of the aforementioned drugs. These observations further underscore the critical importance of having a high index of suspicion for this disease at an early stage and initiating therapeutic intervention as promptly as possible.

## CONCLUSIONS

In summary, we experienced a case of HLH that was diagnosed following surgery for colorectal cancer. Although early intervention is crucial for improving outcomes in M-HLH, its timely diagnosis is often difficult because its clinical presentation closely resembles malignancy-related findings. Clinicians should include cancer-induced HLH in the differential diagnosis of a persistent fever and bicytopenia in patients with advanced solid tumors.
